# Language and Communication in Preschool Children with Autism and Other Developmental Disorders

**DOI:** 10.3390/children8030192

**Published:** 2021-03-04

**Authors:** Vicenç Torrens, Cristina Ruiz

**Affiliations:** Department of Developmental Psychology, National University of Distance Learning, 28040 Madrid, Spain; cristina.ruiz@email.com

**Keywords:** atypical language acquisition, autism, developmental disorders, assessment

## Abstract

In this research, we studied the language and communication skills of preschool children with a diagnosis of autistic syndrome disorder (ASD) (*n* = 51) compared to children with other developmental disorders (DD) (*n* = 42), using direct measures and parental reports when assessing the development of language and communication. As a novelty, this research studied a sample of children with low language and communication skills. We found a high correlation between direct measures and parental reports for both populations. Therefore, we propose that combining the information supplied by direct measures together with that supplied by parental reports would be a suitable strategy for language assessment in these populations. In addition, the results show a delay in language comprehension with respect to language production in children with ASD, along with many difficulties with non-verbal communication, compared to children with other developmental disorders (DD). We also found significant differences between both groups with respect to lexical categories. The differences in language and communication profiles of children with ASD compared to children with other DD might have some implications for diagnoses and language intervention in these populations.

## 1. Introduction

Language difficulties are a crucial symptom in defining the Autism Disorder Spectrum (ASD) since children with this disorder show a deficit in the development of social and communicational interaction [1]. Previous studies have identified differences in the pace of general development, language development, and individual differences between children with ASD and children with typical development [2]. In addition, previous studies on children with ASD have found patterns of language development and gestures in language production and comprehension that are atypical [3,4,5]. The language skills found in older children with ASD are predicted by the early use of gestures [6,7] and early language performance [5,8].

The study of communicative skills in children with ASD presents some difficulties. One such difficulty is the choice of appropriate tools to assess language when children have very limited cognitive and communicative skills. In previous studies on language skills in children with developmental disorders, authors have used direct standardized measures and parental reports. Direct measures might not be suitable when children have very low levels of language comprehension and production [9]. In addition, children who lack motivation or have attentional deficits might show a performance that does not match their real capabilities [10]. Other additional difficulties could be a lack of pragmatic comprehension of the situations to be evaluated, a lack of empathy with the evaluator, environmental distractions, a lack of familiarity with the context in which the assessment takes place, a lack of the ability to point, having to repeatedly answer the same question, low tolerance for frustration, and anxiety in assessment situations [11].

Another way to evaluate language performance in these populations is the use of parental reports in order to complement or replace direct measures. Parental reports also have some limitations, basically because parents have a tendency to overestimate the skills of their own children [12]. In spite of the limitations, though, some researchers have studied the relationship between direct measures and parental reports in early language development, and they have found data in favor of the use of reports by parents when studying communicational skills in children with ASD [4,6]. For instance, strong correlations have been found between parental reports such as Vineland-II (Vineland Adaptive Behavior Scale, VABS-II) [13] and the Mullen Scales of Early Learning (MSEL) [14], with direct measures such as the sequenced inventory of communication development (SICD) [15] when they compared samples of children 2.0 and 3.0 years old diagnosed with ASD, with children diagnosed with other developmental disorders [1]. The MacArthur communicative development inventories (MCDI) [16] are the most common parental reports on early language in research on children with typical or atypical development [17] and are the most common for children with ASD [18]. These reports are surveys on early communicative skills in children with typical development between the ages of 8 and 30 months. In the English version, the surveys have two forms: Words and Gestures, and Words and Sentences, which evaluate early skills for the comprehension and production of words, sentences, grammatical development, imitation, labeling, and gestures. It has also been found that in children with ASD, there are significant correlations between the scores obtained by the MacArthur communicative development inventories and the scores obtained with direct measures and other parental reports. In another study with children with ASD aged between 18 and 33 months, Luyster et al. studied the relationship between MCDI, the VABS-II survey, and the direct measure of MSEL test [6]. Comparing the scores obtained and the equivalent ages, these authors found that all measures correlated significantly, with the production component having the highest correlation. In addition, similar results have been found using these three measures in children with ASD [4,19].

However, many of the previous studies on early communicative skills in children with ASD have not included control groups and/or they have used standardized data in order to compare the results among these populations [2]. The choice of control groups supplies a framework to better understand whether the performance is due to a typical pattern of development or whether this pattern can be classified into a specific group [20]. The evidence available so far suggests that the communicative development in children with ASD differs from the development observed in children with typical development, showing a delayed development in language comprehension and production [2,3,4,5,21]. Further, it has been found that children with ASD have weaker communicative and language skills than children with other DD when matched for chronological and mental age [21,22,23,24,25,26].

Due to the difficulties in evaluating children with no or very limited language skills, children with ASD with very reduced verbal skills have been excluded from many studies [27,28]. One of the most studied aspects in the literature is the fact that children with ASD have a specific language and communicative pattern compared to children with other DD. Some authors have found a delay in language comprehension with respect to language production in children with ASD compared to preschool children with typical development [4,24], or compared to children diagnosed with a non-specified ASD [2], or compared to children with a developmental delay that does not meet the criteria for ASD [2,8], or compared to children with mental retardation [8]. Therefore, finding a delay in language comprehension compared to language production has been considered a sign for diagnosing children with ASD [8].

Another aspect that has been studied in the communicative development of children with ASD is the profile of vocabulary based on semantic and grammatical categories. The results are not conclusive because it is difficult to compare all studies due to differences in the designs and methods, the levels of development of participants, and the diagnosis criteria of the samples [29]. When authors compared the lexicon of children with ASD and children with typical development, the results were contradictory: Bruckner et al. found differences in the lexicon for vocabulary in comprehension in children with ASD compared to children with typical development when analyzing the items of MCDI [30]. However, when comparing children with ASD and children with typical development, Charman et al. found that the vocabulary pattern of preschool children was similar [3]; in the same vein, when comparing the lexicon of children aged 2.0 and 3.0 years, Weismer et al. found that the expressive vocabulary of children with ASD was delayed but similar to the vocabulary found in late talkers, which is evidence that differences were quantitative more than qualitative [29]. Luyster et al. could not find any differences when comparing the vocabulary of children with ASD with children with typical development and mentally retarded children, with respect to some categories of MCDI such as nouns, predicates, and pivot words [8]. However, Tager-Flusberg et al. found that children with ASD used more nouns, whereas children with Down’s syndrome used more pivot words (especially pronouns or determiners) when comparing children with Down’s syndrome and children with ASD in a longitudinal study [31]. With respect to semantic development in children with ASD and children with typical development, many differences have been found in semantic categorizing and integration between these populations [32,33].

The use of gestures is another aspect under study with respect to communicative development in children with ASD. It has been found that children with ASD experience a delay in non-verbal communication compared to children with typical development [3,5,21,34]. Also, differences have been found when comparing the use of gestures in children with ASD and children with mental retardation. For example, Sigman and Ungerer found that the ability to imitate gestures is lower in children with ASD compared to children with mental retardation and children with typical development. In this vein, it has been found that children with Down’s syndrome are more advanced in their development of gestures compared to the development of language comprehension in children with typical development [35]. Toret and Acarlar compared the development of gestures in children with ASD, children with Down’s syndrome, and children with typical development, and they found differences in the frequency of gestures: children with typical development used gestures more frequently than the rest of the groups; children with ASD used gestures the least [34].

Based on the facts presented in this section, this research tries to study the communicative skills of preschool children aged between 2.0 and 6.0 years who have been diagnosed with ASD and who have low language skills. We have compared the communicative development of children with ASD and children with other DD. Previous studies have only compared communicative skills with children younger than 3.0 years old [2,8], or they have used samples with an age range that is too broad, such as a sample aged between 1.0 and 11.0 years [24]. The comparison between both groups can allow us to conclude whether all developmental disorders have the same language and communication pattern or whether these have different profiles.

Another goal was to explore the relationship between the scores of parental surveys and the scores of standardized tests, which have a direct measure, in order to check their external validity. Our prediction is that children with ASD will perform less well in linguistic and communicative skills than children with other DD and that children with ASD will perform less well in language comprehension with respect to production, which is the opposite pattern than that found in children with other DD or with typical development. With respect to the use of gestures, we expect to find a lower performance in children with ASD compared to children with other DD. Finally, we expect to find different language profiles with respect to vocabulary and semantic profiles within these populations.

## 2. The Present Study

### 2.1. Participants

The sample in this study is based on the database of two studies developed by the team of Research on Autism and Developmental Disorders, which is based at Stanford University in California, which aimed to measure the effectiveness of the pivotal response treatment (PRT) on language skills in children with developmental disorders [36]. Participants were preschool children aged between 2.0 and 5.11 years who lived in the San Francisco Bay Area, California. The first group (ASD, *n* = 51, mean age = 47.33 months, boys = 89%, girls = 11%) were children with ASD. The second group (DD, *n* = 42, mean age = 42.07 months, boys = 58%, girls = 42%) were children with other DD who did not meet the criteria to be diagnosed with ASD. The second group was very heterogeneous (unspecified developmental delay (*n* = 12), developmental language disorder (*n* = 6), Down’s syndrome (*n* = 6), cerebral palsy (*n* = 6), cri-du-chat syndrome (*n* = 6), Klinefelter syndrome (*n* = 3), and fragile X syndrome (*n* = 3)). This study has been approved by the Ethics Committee of the National University of Distance Learning, with the reference COEDU_FECORA. The protocol of the Ethics Committee of the National University of Distance Learning was approved on 7th May 2018. With respect to the ethnicity of participants, 70% of participants were White, 14% were Hispanic, 10% were African American, and 6% were Asian. With respect to languages spoken, 80% were monolingual English speakers, 14% were bilingual Spanish/English speakers, and 6% were Chinese/English bilingual speakers. All participants were middle class; ethnicity and language dominance was balanced among the research groups. All parents of the participants included in the sample provided written informed consent at the beginning of the study, and pertinent measures have been followed to maintain their anonymity.

### 2.2. Procedure

In the autism group (ASD), the participants were recruited with the following criteria: they had to have a diagnosis of ASD based on the revised version of the Autism Diagnostic Interview (ADI-R) [37], Autism Diagnostic Observation Schedule (ADOS) [38], and the opinion of a clinical expert. In addition to this, participants had to present a delay in the acquisition of language of at least one standard deviation under the mean for language production of the Preschool Language Scales 5th edition (PLS-5) [39]. In the second group of other DD, the criteria for inclusion were a diagnosis of mental retardation or language impairment based on DSM-IV-TR, CIE-10, and the evaluation of a clinical expert. In addition to this, participants needed to have a delay in the acquisition of language of at least one standard deviation under the mean for language production of the Preschool Language Scales 4th edition (PLS-4) [40]. For both groups, parents had to complete different surveys, such as Word and Gestures from MCDI and VABS-II scales. In addition, when parents visited the lab, the following tests were supplied: MSEL scales and Preschool Language Scales (PLS-4) [39,40]. In this study, we used the results obtained in the baseline for each of the researches. We did not collect any qualitative information from the parents aside from survey responses to MCDI and VABS-II scales.

### 2.3. Materials

With respect to cognitive development, we ran the MSEL scales [14]. The score for non-verbal IQ was obtained through the subtests of visual organization and motor skills. With respect to language development, we ran the MCDI parental report, which has two sections: the survey Words and Gestures, and the survey Words and Sentences [16]. The survey Words and Gestures has two sections: the first section measures language comprehension, labeling of objects, and imitation. Words is organized into 19 categories, which consist of nouns, sounds of animals, words for actions, words for timing, descriptive words, pronouns, interrogative words, prepositions, places, and quantifiers. Words in these categories can be classified as closed words or open words. Open words can be nouns, verbs, and adjectives; on the other hand, closed words can be pronouns, determiners, conjunctions, prepositions, and some adverbs, and it is a category to which it is difficult to add new terms; different styles of development are characterized by a different pattern in the development of these categories. Open words mean that new words can be added to the class as the need arises. The classes of nouns, verbs, and adjectives are potentially infinite since they can continually increase in the process of lexical acquisition. Open words can appear as lone words in a sentence, and they can combine with other open or closed words. On the other hand, closed words is a category to which it is difficult to add new terms (i.e., pronouns, determiners, conjunctions, prepositions, and some adverbs) since they are made up of finite sets of words. Closed words never appear as lone words in a sentence, and they are never combined with other closed words. Closed words are usually more difficult to learn since they usually have syntactic functions, such as specifying the sex and number of nouns, defining the function of a complement in a sentence, or gathering different sentences. In the parental report MCDI, the category of prepositions and places includes prepositions and adverbs of place. The category of Words for time includes basically temporal adverbs. In English, adverbs can be both open and closed words; more precisely, some adverbs of place and time, such as the ones included in MCDI, are closed class; this issue will be discussed in the Discussion section. The second section evaluates the use of gestures and consists of a list of 63 gestures organized into two sections: early gestures (e.g., Communicative Gestures, Games, and Routines) and late gestures (e.g., Actions with Objects, Pretending to be a Parent, and Imitating Adult Actions). As a second evaluation, we ran VABS-II [13], MSEL [14], and PLS scales [39,40], which are standardized tests.

### 2.4. Design

We used data from all surveys and tests described for both groups of participants. We tested the relation between direct measures on language production and comprehension for all tests for each sample using a Spearman correlation. In order to analyze the relations of the samples, we excluded the analysis of the Preschool Language Scale because we used a different version for each group. Then, we analyzed the language profiles within each group using equivalent ages for language comprehension and production for every group in the MSEL, VABS-II, and PLS tests in order to compare these scores with the chronological age and to compare the performance in language comprehension and production within each group. Because of the reduced number of participants, we used the non-parametric Wilcoxon test.

Afterward, we compared the direct measures between the groups for language comprehension and production. We used a non-parametric test for independent measures (i.e., the Mann–Whitney U test). Then, we studied the differences in communicative skills for MCDI between both ASD and DD groups. The analyses were applied on seven variables: the first variable was communicative skills before speech, which was calculated from five items of subtests, first signs of understanding, and starting to talk; this grouping was first used by Luyster et al. [5]. The second variable was the number of sentences (up to 28) that parents indicated their children could understand. The third and fourth variables consisted of the number of words understood and produced by children (up to 396). The fifth and sixth variables were the total number of early and late gestures following the distinction proposed by the authors of MCDI [16]. Finally, we analyzed the differences between groups with respect to the kind of vocabulary for language comprehension and production reported by parents. Nine participants in the ASD group and three of the DD group did not have any language production, and therefore we did not apply any sort of analysis for it. We explored the differences between the grammatical categories with respect to the total vocabulary of children in MCDI and then analyzed the semantic categories of nouns used compared to the proportions of each of the total of nouns for lexical comprehension and production.

## 3. Results

### 3.1. Relations between Language Measures

First of all, we compared the performance of the scores of MCDI for vocabulary and the direct measures of VABS-II and MSEL tests for both language comprehension and production, collapsing both groups: for MCDI and VABS surveys, the correlations scored the values *ρ* = 0.608, *p* < 0.002 for language comprehension and the values *ρ* = 0.795, *p* < 0.002 for language production; for MCDI and Mullen test, the correlations scored the values *ρ* = 0.462, *p* < 0.02 for language comprehension and the values *ρ* = 0.872, *p* < 0.002 for language production; for VABS and MSEL test, the correlations scored the values *ρ* = 0.57, *p* < 0.02 for language comprehension and the values *ρ* = 0.705, *p* < 0.02 for language production. For all the analyses mentioned above, we applied the Bonferroni correction for inflated alpha levels. When we analyzed the samples within each group separately, we included the scores of PLS-4 and PLS-5. However, this test was not included in the analysis of the total sample, as mentioned before, since we used different versions for each group. In the ASD group (*n* = 51), we found significant correlations between all measures from moderate to high. However, for the other DD group (*n* = 42), the pattern was different: with respect to language production, there were correlations for all measures except for PLS-4 and VABS-II; with respect to language comprehension, we did not find significant correlations between parental reports and direct measures, which was perhaps because the sample was very heterogeneous. However, we found correlations within the parental reports (VABS-II and MCDI) and within direct measures (MSEL and PLS4). Results are shown in Table 1.

### 3.2. Language Profiles within Each Group

We applied the non-parametric Mann–Whitney U test to check whether there were differences between groups with respect to chronological age and non-verbal IQ. We did not find any significant difference for any of the variables and therefore did not include any of these in the analysis between groups. However, we compared the equivalent ages obtained for language comprehension and production for MSEL, PLS, and VABS-II, with respect to chronological age (see Table 2), and the data showed a lower performance to be expected for chronological age for all measures and for all groups, with significant differences (*p* < 0.001).

Table 3 shows the descriptive statistics corresponding to equivalent ages for language comprehension and production for MSEL, VABS-II, and PLS, with standard deviations, showing a high variability for all areas evaluated. In order to study all communicative profiles for each group, we analyzed the differences between the scores for equivalent ages in language comprehension and production for the three measures. With respect to the DD group, the performance average was higher in language comprehension than in language production, although when we applied the non-parametric test, we found no significant differences between language comprehension and production for any of the areas explored (see Table 1). With respect to the ASD group, the average of equivalent ages was higher for language production than language comprehension in the MSEL and PLS-5 tests, whereas in the VABS-II parental report, the age average was higher for language comprehension. In this group, we did not find any significant difference in equivalent ages between language comprehension and production. When we compare the size of the effect, we can see in Table 3 that the difference of means between language comprehension and production is higher for the DD group than the ASD group.

### 3.3. Differences between Groups

Table 4 shows the descriptive statistics (mean and standard deviation) for language comprehension and production, the effect of size for the differences between groups, and the significance obtained for each variable using the Mann–Whitney U test.

In order to analyze the differences in language comprehension and production between groups, we compared the direct measures for MSEL and VABS-II. We also compared performance in word comprehension and production for MCDI and sentence comprehension for each group. With respect to language comprehension, we found significant differences for all variables, with the highest score average in the DD group for the two standardized tests (MSEL (z = −2.102, *p* = 0.04) and VABS-II (z = −3.259, *p* = 0.001)) and the survey MCDI: vocabulary comprehension total (z = −1.061, *p* = 0.289) and sentence comprehension (z = −2.222, *p* = 0.026). However, with respect to language production, none of the analyses showed any significant differences for any variable analyzed.

Afterward, we analyzed the differences between groups with respect to pre-speech skills: the analysis showed no significant differences between groups with respect to skills previous to speech for MCDI (z = −1.061, *p* = 0.289). With respect to the differences in language comprehension and production in the MCDI categories, we compared the proportion of the number of words for each category with respect to total vocabulary for each participant. In vocabulary comprehension (see Table 5), we observed significant differences for prepositions, where the highest proportion was in the DD group (z = −2.866, *p* = 0.004). We could not find any significant differences in the rest of the categories. With respect to language production (Table 6), we found significant differences in the proportions of the following three categories, with the highest scores in the DD group: words related to time (z = −3, 03, *p* = 0.002), pronouns (z = −2.193, *p* = 0.028) and prepositions (z = −2.928, *p* = 0.003). Further, we found significant differences between groups with respect to the proportions of adjectives in the total sample lexical production and, this time, the ASD group obtained the highest proportions (z = 2.284, *p* = 0.022). With respect to the analysis of the semantic categories of nouns, we could not find any significant differences in the proportions of any of the categories with respect to the total number of nouns that are part of lexical comprehension and production in children, following the information supplied by parents.

With respect to the use of gestures (see Table 7), the results show significant differences between groups, with higher scores for the DD group regarding total score (z = −3.001, *p* = 0.003), in early gestures (z = −3.41, *p* = 0.001), and late gestures (z = −3.001, *p* = 0.003).

## 4. Discussion

### 4.1. Relations between Language Measures

After fully analyzing the data of the sample, including the children with ASD and DD, we found significant correlations between all measures with respect to the scores in language comprehension and production. The highest correlations were in language production. We observed the same pattern of correlations when analyzing children with ASD and children with other DD. These results replicate the relations found in previous studies [2,6,19]; as a novelty, this research studied these correlations in a sample of children with low verbal skills. Children with ASD showed high significant correlations both in direct measures and parental reports. These results show evidence in favor of the use of parental reports in the study of communicative development in children with ASD with low verbal skills since these reports are significantly related to direct measures and standardized tests.

With respect to the group of children with other DD, the significant correlations between parental measures and language proficiency tests were restricted to all correlations except for PLS-4 and VABS-II on language production and restricted to direct measures (MSEL and PLS) and parental reports (MCDI and VABS-II) on language comprehension. Following these results, we can conclude that direct measures and parental reports offer differentiated information, depending on when we study language comprehension in children with other DD. Previous studies found that it is very difficult to assess language comprehension in children with communicative difficulties since the conditions where the assessment takes place [11] or the motivational and attentional aspects [10] make it difficult to observe the capacities of language comprehension. Other studies found no weaker agreement when assessing language comprehension, and they found that parent reports of language skills were equivalent to scores on direct testing in language comprehension [41]. Miller et al. argue that it might be due to their reliance on Vineland, which is a semi-structured parent review, instead of a parent report checklist; this outcome suggests to these authors that parents are usually reliable reporters. Taking into account parental reports, it has also been observed in previous studies that parents overestimate the skills in language comprehension of their own children [12]. Some authors suggest that parents usually report higher fine motor skills compared to direct assessment; this could be due to the fact that parents assume that children can perform a motor task without having observed it; in addition, children might not want to perform some tasks during assessment because they are not interested in the testing materials or because they have difficulties comprehending testing demands [41]. Another reason for the discrepancy is the fact that a child might not perform an item during a direct assessment, but she might perform that item at home. The discrepancy between these language measures could reflect the fact that the assessment by parents of their own children differs from the assessment by expert evaluators, although we should be careful because of the heterogeneity and the size of the samples. However, based on the results of this study, we can conclude that a suitable strategy for language assessment would be to combine the information supplied by direct measures together with the information supplied from parental reports [9].

### 4.2. Language Profiles within Each Group

With regard to the language profiles for each group, after comparing the scores expected for their age on the standardized measures, the results show a delay in language skills for both groups. This is not a surprise since communicative and language difficulties are basic symptoms in ASD and developmental language disorder [1]. In addition, one of the conditions to be part of the samples in this study was to have a language delay.

In order to find out whether there are different language profiles for both groups, we compared the scores for typical children with equivalent ages for language comprehension and production. In the DD group, we found a typical language pattern, where language comprehension skills were more developed than language production skills. With respect to children with ASD, we expected to find the opposite pattern since previous studies have found differences in this direction [24]. In fact, we found that in the parental report of VABS-II, the mean of the scores was higher for language production than language comprehension. With respect to the rest of the language measures (MSEL and PLS-5), we found the same pattern of higher scores in language production but with no significant differences. In any case, we found a delay in language comprehension with respect to language production for children with ASD if we compare the scores with the group of children with other DD; this delay could be due to the difficulties of children with ASD to generalize skills across contexts [42]. However, some previous studies did not find a significant interaction between language measures and the DD group [41]. If we take a look at the size of the effect of the differences between language comprehension and production, we find that the size of the effect is higher for children with other DD. In addition, when we compared the direct measures for language comprehension in different measures, we observed significant differences in both groups for all variables in language comprehension, with higher performance in children with other DD; however, we found no significant differences in language production for none of the measures. Therefore, both groups have a similar performance in language production, but difficulties in language comprehension were greater for children with ASD [3,4,5]. Another aspect that could have been taken into account is the fact that some skills could have a reporter bias: it has been found in previous studies that there is a lower correlation for the assessment of language comprehension compared to language production [12,43]. Even though we did not test this fact in this study, previous studies have found mixed results: while there is a high correlation of items measuring basic skills, it is not the case for more demanding language skills [41].

### 4.3. Differences between Groups in Language Skills

When we compare language skills between both groups, we find that the levels of language comprehension differ significantly between groups, whereas the levels of language production are similar between both groups. With respect to language comprehension, we found significant differences for all the analyzed variables, that is, the performance in language comprehension in direct measures, Mullen and PLS with the parental report VABS-II, and with sentence comprehension and vocabulary size of MCDI.

With respect to the properties of vocabulary based on the categories proposed for MCDI, the results show that language comprehension is very similar for both groups. In fact, when we compare the proportions for each category of word with respect to the total number of words understood, we found no difference, except for prepositions, since the group of children with other DD had higher proportions of these categories. With respect to language production, the distribution of words based on categories for both groups is similar, except for the fact that children with other DD have a higher proportion of language production for prepositions, pronouns, and words for time. Therefore, the results obtained when we compare these samples supply more evidence that favors the results found by Tager-Flusberg et al. [31] about the tendency for children with Down’s syndrome to use more closed words than do children with ASD, since we found that the group of children with other DD had higher proportions for prepositions and places than the group of children with ASD. Finally, in language production, we found that children with ASD used more adjectives proportionally than children with other DD, which provides more evidence for the existence of different patterns of lexical categories in language production in children with ASD, with respect to children with other DD regarding the difference between open and closed words. This different pattern could be due to different cognitive styles among these populations: Nelson found that expressive children use a higher rate of pronouns than referential children, who are more focused on the learning of full noun phrases and words; this difference is higher when children have a low MLU (i.e., in the initial stages of language acquisition) [44]. She also found that referential children produce more subjects with the thematic role of agent, whereas referential children use more subjects with the thematic role of experiencer. Nelson found that referential children use more qualifying adjectives, whereas referential children use more possessive adjectives. According to Nelson, referential children start to learn lexical items and afterward learn the parameters concerning phrase structure, whereas expressive children learn patterns of word order and then they increase their lexical repertoire. Lieven found that referential children used sentences with less variability than expressive children [45,46]. Also, she found that referential children are more analytical since they learn lexical items with fewer complements and specifiers than expressive children, whereas expressive children include more components in their phrases, and these forms are less analyzed. Bates et al. also found that expressive children had more social skills and better memory storage than referential children, whereas referential children were more analytical [47]. She proposes that the individual differences found in language development depend on faculties such as analytical processing versus memory skills and the performance on language production versus performance on language comprehension. Therefore, it could be the case that children with ASD could have a referential cognitive style compared to children with other DD, who could have a more expressive style.

We then compared the semantic categories of nouns from the lexical sample of both groups found in the parental reports. However, in this case, we did not find any significant difference in the proportions observed for either lexical comprehension or language production between both children with ASD and children with other DD.

### 4.4. Differences between Groups on Non-Verbal Communication

Finally, we analyzed the differences in non-verbal communication between both groups, and our results provide evidence in favor of the higher performance of children with other DD, because we found significant differences in the use of gestures with respect to children with ASD, based on parental reports. It could be the case that the lower use of gestures in ASD is related to their communication difficulties in this population and with better communication abilities in Down’s syndrome. Even though it only applies to a subgroup of the children with DD, previous studies found that children with Down’s syndrome are more advanced in their development of gestures compared to children with typical development [35]. Caselli et al. studied the development of language and communication in children with Down’s syndrome. The goal of this research was to examine the relations between language comprehension, language production, and the development of gestures in children with Down’s syndrome compared to typically developing children. They found that children with Down’s syndrome had a lower performance compared to typically developing children in language development. They found a similar development between lexical comprehension and the development of gestures. However, they found that children with Down’s syndrome had a higher gestural development compared to typically developing children [35]. They found that children with Down’s syndrome produce a higher frequency of symbolic communicative gestures, pretending gestures, and actions to perform symbolic transformations. Following Caselli et al., in the initial stages, the gestural and vocal production of children with Down’s syndrome are similar to those of typically developing children matched for word comprehension; however, they found that later on, symbolic communicative gestures and actions increase and are more developed in children with Down’s syndrome, based on their level of development of word comprehension and production. This fact could explain the data that we have obtained in our study so far.

## 5. Conclusions

The results found in this research might have implications for the assessment of children with low language and communication skills: the consistency between different measures supports the use of direct measures and parental reports for therapists working with children with ASD and other DD. The specific patterns found in the delay in the development of language comprehension, the properties of vocabulary, and the low use of gestures of children with ASD compared to children with other DD could help practitioners with a differential diagnosis after a deeper exploration from a clinical perspective. The results found in this research underline the importance of including improvements in verbal and non-verbal communication in children with ASD as important goals on intervention. However, we should be cautious because we did not collect any qualitative information from the parents aside from survey responses to MCDI and VABS-II scales, also because there was a high heterogeneity of the participants, and because the size of the sample was small in this study, which could make it difficult to generalize the results to other studies and to provide a complete profile of the properties of language and communication in these populations.

## Figures and Tables

**Table 1:** Spearman correlations between measures of language.

ASD	*ρ*	*p*	DD	*ρ*	*p*
**Comprehension**					
MCDI-VABS	0.656	*p* < 0.001	MCDI-VABS	0.765	*p* < 0.001
MCDI-MSEL	0.512	*p* < 0.001	MCDI-MSEL	0.097	NS
MCDI-PLS	0.511	*p* < 0.001	MCDI-PLS	0.054	NS
PLS-VABS	0.645	*p* < 0.001	PLS-VABS	0.243	NS
MSEL-VABS	0.636	*p* < 0.001	MSEL-VABS	0.037	NS
MSEL-PLS	0.712	*p* < 0.001	MSEL-PLS	0.709	*p* < 0.001
**Production**					
MCDI-VABS	0.831	*p* < 0.001	MCDI-VABS	0.798	*p* < 0.001
MCDI-MSEL	0.889	*p* < 0.001	MCDI-MSEL	0.840	*p* < 0.001
MCDI-PLS	0.782	*p* < 0.001	MCDI-PLS	0.544	*p* < 0.001
PLS-VABS	0.724	*p* < 0.001	PLS-VABS	0.496	NS
MSEL-VABS	0.698	*p* < 0.001	MSEL-VABS	0.777	*p* < 0.001
MSEL-PLS	0.751	*p* < 0.001	MSEL-PLS	0.716	*p* < 0.001

Note: MCDI: MacArthur Communicative Development Inventories [16]. VABS: Vineland Adaptive Behavior Scale [13]. MSEL: Mullen Scales of Early Learning [14]. PLS: Preschool Language Scales [39,40]. *p*: statistical significance.

**Table 2:** Differences for equivalent ages for MSEL, PLS, and VABS-II in language comprehension and production compared to chronological age.

ASD	*U*	*p*	DD	*U*	*p*
**MSEL** (*n* = 51)			**MSEL***(n* = 42)		
Comprehension	17. 77	*p* < 0.001	Comprehension	7.72	*p* < 0.001
Production	15.77	*p* < 0.001	Production	10.81	*p* < 0.001
**PLS-5** (*n* = 51)			**PLS-4** (*n* = 42)		
Comprehension	19.04	*p* < 0.001	Comprehension	8.15	*p* < 0.001
Production	19.52	*p* < 0.001	Production	8.28	*p* < 0.001
**VABS-II** (*n* = 51)			**VABS-II** (*n* = 42)		
Comprehension	19.34	*p* < 0.001	Comprehension	24.04	*p* < 0.001
Production	18.71	*p* < 0.001	Production	8.22	*p* < 0.001

Note: MSEL: Mullen Scales of Early Learning [14]. PLS: Preschool Language Scales (PLS-4) [39,40]. VABS-II: Vineland Adaptive Behavior Scale [13]. *p*: statistical significance.

**Table 3:** Descriptive statistics for equivalent age on language comprehension and production for all measures of language MSEL, VABS-II, and PLS.

			Comprehension			Production		Size of Effect		Contrast Comprehension and Production	
	n		Median	M	SD	Median	M	SD	*d*	Z	*p*
ASD	51	MSEL	14.5	17.33	7.73	16.5	17.41	8.98	−0.01	0.396	*p* > 0.05
	51	PLS-5	16	17.7	6.32	18	17.98	6.16	−0.04	−0.45	*p* > 0.05
	50	VABS-II	15	15.96	6.79	15.5	15.72	7.03	0.04	−0.245	*p* > 0.05
DD	42	MSEL	22	21.46	9.14	16	16.69	6.42	0.6	−2.125	*p* < 0.05
	42	PLS-4	25	23.77	8.77	21	20.23	3.68	0.53	−1.06	*p* > 0.05
	42	VABS-II	23	25.92	12.98	19	19	5.15	0.7	−1.69	*p* > 0.05

Note. MSEL: Mullen Scales of Early Learning [14]. VABS-II: Vineland Adaptive Behavior Scale [13]. PLS: Preschool Language Scales [39,40]. M: mean. SD: standard deviation. *d*: *d* Cohen statistics for size of effect. Z: statistics for contrast U Mann–Whitney. *p*: statistical significance.

**Table 4:** Descriptive statistics corresponding to direct measures in language comprehension and production in MSEL, VABS-II, PLS, and MCDI and differences between groups.

Test	ASD		DD		ASD vs. DD	
	M	SD	M	SD	*d*	Z
**MSEL**						
Comprehension	18.10	6.63	22.77	6.66	−0.7	(z = −2.102, *p* = 0.04)
Production	16.96	7.52	17.54	4.86	−0.09	(z = −0.401, *p* = 0.69)
**PLS**						
Comprehension	22.18	5.58	27.50	6.89	−0.85	(z = −2.608, *p* = 0.009)
Production	22.73	5.19	25.57	3.30	−0.67	(z = −1.905, *p* = 0.057)
**VABS-II**						
Comprehension	15.18	5.98	22.00	5.91	−1.15	(z = −3.259, *p* = 0.001)
Production	24.75	11.71	30.15	10.87	−0.48	(z = −1.721, *p* = 0.085)
**MCDI**						
Comprehension	181.12	114.25	223.93	99.83	−0.4	(z = −1.061, *p* = 0.289)
Production	94.45	99.03	62.99	97.84	0.32	(z = 0.823, *p* = 0.410)
Pre-speech skills	3.55	1.40	4.29	0.99	−0.62	(z = −1.783, *p* = 0.075)
Sentence comprehension	16.20	8.22	21.57	5.12	−0.8	(z = −2.222, *p* = 0.026)

Note. MSEL: Mullen Scales of Early Learning [13]. VABS-II: Vineland Adaptive Behavior Scale [12]. PLS: Preschool Language Scales (PLS-4: [38,39]). M: mean. SD: standard deviation. *d*: *d* Cohen statistics for size of effect. Z: statistics for contrast U Mann–Whitney. *p*: statistical significance.

**Table 5:** Descriptive statistics corresponding to the proportions for each MCDI category with respect to lexical comprehension.

Test	ASD		DD			ASD vs. DD
MCDI	M	SD	M	SD	*d*	Z, *p*
**Comprehension**						
Categories of MCDI						
Nouns	0.69	0.08	0.68	0.04	0.17	(z = 0.527, *p* = 0.598)
Verbs	0.17	0.05	0.15	0.02	0.57	(z = 0.997, *p* = 0.319)
Time	0.01	0.01	0.004	0.01	0.6	(z = 0.578, *p* = 0.563)
Adjectives	0.05	0.03	0.06	0.02	−0.4	(z = −0.950, *p* = 0.342)
Pronouns	0.01	0.01	0.01	0.01	0	(z = −1.94, *p* = 0.052)
Interrogatives	0.01	0.01	0.004	0.005	0.8	(z = −0.327, *p* = 0.744)
Prepositions	0.02	0.01	0.03	0.02	−0.67	(z = −2.866, *p* = 0.004)
Quantifiers	0.01	0.01	0.01	0.01	0	(z = −0.68, *p* = 0.496)
Sounds	0.05	0.03	0.05	0.02	0	(z = −0.743, *p* = 0.458)

Note: MacArthur Communicative Development Inventories [16]. M: mean. SD: standard deviation. *d*: *d* Cohen statistics for size of effect. Z: statistics for contrast U Mann–Whitney. *p*: statistical significance.

**Table 6:** Descriptive statistics corresponding to the proportions for each category of MCDI with respect to lexical production.

Test	ASD			DD		ASD vs. DD
MCDI Production	M	SD	M	SD	*d*	Z, *p*
Categories of MCDI						
Nouns	0.19	0.19	0.15	0.17	0.22	(z = 0.144, *p* = 0.886)
Verbs	0.11	0.08	0.07	0.05	0.61	(z = 1.687, *p* = 0.092)
Time	0.001	0.004	0.04	0.05	−1.44	(z = −3.03, *p* = 0.002)
Adjectives	0.03	0.04	0.01	0.02	0.67	(z = 2.284, *p* = 0.022)
Pronouns	0.01	0.02	0.02	0.02	−0.5	(z = −2.193, *p* = 0.028)
Interrogatives	0.002	0.004	0.0003	0.001	0.65	(z = 1.389, *p* = 0.165)
Prepositions	0.02	0.03	0.06	0.07	−0.8	(z = −2.928, *p* = 0.003)
Quantifiers	0.01	0.02	0.02	0.02	−0.5	(z = −0.833, *p* = 0.405)
Sounds	0.08	0.16	0.09	0.05	−0.09	(z = −1.933, *p* = 0.053)

Note: MacArthur Communicative Development Inventories [16]. SD: standard deviation. *d*: *d* Cohen statistics for size of effect. Z: statistics for contrast U Mann–Whitney. *p*: statistical significance.

**Table 7:** Descriptive statistics corresponding to direct measures for early gestures, late gestures, and the total number for MCDI.

Test	ASD		DD			ASD vs. DD
	M	SD	M	SD	*d*	Z, *p*
**MCDI** **Gestures**						
Early	9.47	3.97	13.71	3.2	−1.17	(z = −3.41, *p* = 0.001)
Late	21.14	9.63	30.07	11.42	−0.84	(z = −2.659, *p* = 0.008)
Total	30.47	12.38	43.79	13.92	−1.01	(z = −3.001, *p* = 0.003)

Note: MacArthur Communicative Development Inventories [16]. SD: standard deviation. *d*: *d* Cohen statistics for size of effect. Z: statistics for contrast U Mann–Whitney. *p*: statistical significance.
